# RNA packaging into extracellular vesicles: An orchestra of RNA‐binding proteins?

**DOI:** 10.1002/jev2.12043

**Published:** 2020-12-28

**Authors:** Fabrizio Fabbiano, Jessica Corsi, Elena Gurrieri, Caterina Trevisan, Michela Notarangelo, Vito G. D'Agostino

**Affiliations:** ^1^ Department of Cellular Computational and Integrative Biology (CIBIO) University of Trento Trento Italy

**Keywords:** extracellular vesicles, EVs, inhibitors, protein domains, RBPs, RNA, RNA‐binding proteins, RNA consensus

## Abstract

Extracellular vesicles (EVs) are heterogeneous membranous particles released from the cells through different biogenetic and secretory mechanisms. We now conceive EVs as shuttles mediating cellular communication, carrying a variety of molecules resulting from intracellular homeostatic mechanisms. The RNA is a widely detected cargo and, impressively, a recognized functional intermediate that elects EVs as modulators of cancer cell phenotypes, determinants of disease spreading, cell surrogates in regenerative medicine, and a source for non‐invasive molecular diagnostics. The mechanistic elucidation of the intracellular events responsible for the engagement of RNA into EVs will significantly improve the comprehension and possibly the prediction of EV “quality” in association with cell physiology. Interestingly, the application of multidisciplinary approaches, including biochemical as well as cell‐based and computational strategies, is increasingly revealing an active RNA‐packaging process implicating RNA‐binding proteins (RBPs) in the sorting of coding and non‐coding RNAs. In this review, we provide a comprehensive view of RBPs recently emerging as part of the EV biology, considering the scenarios where: *(i)* individual RBPs were detected in EVs along with their RNA substrates, *(ii)* RBPs were detected in EVs with inferred RNA targets, and *(iii)* EV‐transcripts were found to harbour sequence motifs mirroring the activity of RBPs. Proteins so far identified are members of the hnRNP family (hnRNPA2B1, hnRNPC1, hnRNPG, hnRNPH1, hnRNPK, and hnRNPQ), as well as YBX1, HuR, AGO2, IGF2BP1, MEX3C, ANXA2, ALIX, NCL, FUS, TDP‐43, MVP, LIN28, SRP9/14, QKI, and TERT. We describe the RBPs based on protein domain features, current knowledge on the association with human diseases, recognition of RNA consensus motifs, and the need to clarify the functional significance in different cellular contexts. We also summarize data on previously identified RBP inhibitor small molecules that could also be introduced in EV research as potential modulators of vesicular RNA sorting.

## 1 INTRODUCTION

Extracellular vesicles are cell‐secreted membranous particles showing heterogeneous size and molecular composition. EVs can originate from multi‐vesicular bodies (MVBs), giving rise to vesicles referred to as exosomes, or from the plasma membrane budding, in this case referred to as microvesicles (MVs) or ectosomes (Yáñez‐Mó et al., 2015). The mean diameter of EVs ranges from nanometres to few micrometres, and the variety of their biological content includes lipids, proteins, and nucleic acids. It is now widely recognized that cells secrete EVs as vehicles to communicate, exchanging factors that can modulate cell signalling pathways as well as transcriptional and post‐transcriptional processes (Sork et al., 2018). Indeed, seminal studies indicated that alterations in EV abundance and quality might influence the phenotype of tumour cells, the cross‐talk with immune cells, and the spreading of the tumour in different organs (Becker et al., 2016; Choi et al., 2017; Cianciaruso et al., 2019). Moreover, EVs are also taking place in the field of regenerative medicine, acting as cell surrogates in a‐cellular biocompatible scaffolds (Cabral et al., 2018), or in targeted therapy, acting as modifiable cell‐derived vectors (Armstrong et al., 2017). Nevertheless, the role of EV‐RNA is also emerging in liquid biopsy, promising as an alternative non‐invasive source for molecular testing, possibly complementing the cell‐free DNA and circulating tumour cells (CTCs) (Heitzer et al., 2019).

The RNA is part of the EV cargo and the focus of many functions ascribed to EVs. Currently, the Vesiclepedia database includes more than 27,000 entries for mRNAs and more than 10,000 entries for non‐coding RNAs (http://microvesicles.org). The expansion of our knowledge on the different classes of RNAs populating the EV cargo, including coding and non‐coding species (spliceosomal RNAs, lncRNAs, Y RNAs, tRNA‐derived small RNAs, miRNAs, tiny transcription‐initiation RNAs, promoter‐associated short RNAs, termini‐associated short RNAs, TASRs, antisense TASRs, 3ʹUTR‐derived RNAs, and tRNA‐derived short RNAs) results from the recent broad applicability of sensitive RNA detection technologies and computational tools. However, we are still far from predicting a specific RNA sorting into bulk or sub‐populations of EVs according to the cell physiology.

Very recently, the activity of competitive RNA‐binding proteins is emerging as a crucial determinant in diversifying the enrichment of selected transcripts into EVs. Interestingly, the application of biochemical, cell‐based, and computational approaches revealed in several models the enrichment of transcript sequences, defining ‘RNA motifs’, recurrently found in EVs and validated as consensus substrate for RNA‐binding proteins (RBPs). In this review, we provide a comprehensive view of the current knowledge about RBPs described as part of the EV biology. We included the scenarios where *(i)* individual RBPs were detected in EVs along with their substrate RNAs, *(ii)* RBPs were detected in EVs but the substrate RNA targets are inferred only, and *(iii)* transcripts found in EVs which harboured sequence motifs mirroring the activity of RBPs.

**FIGURE 1:**
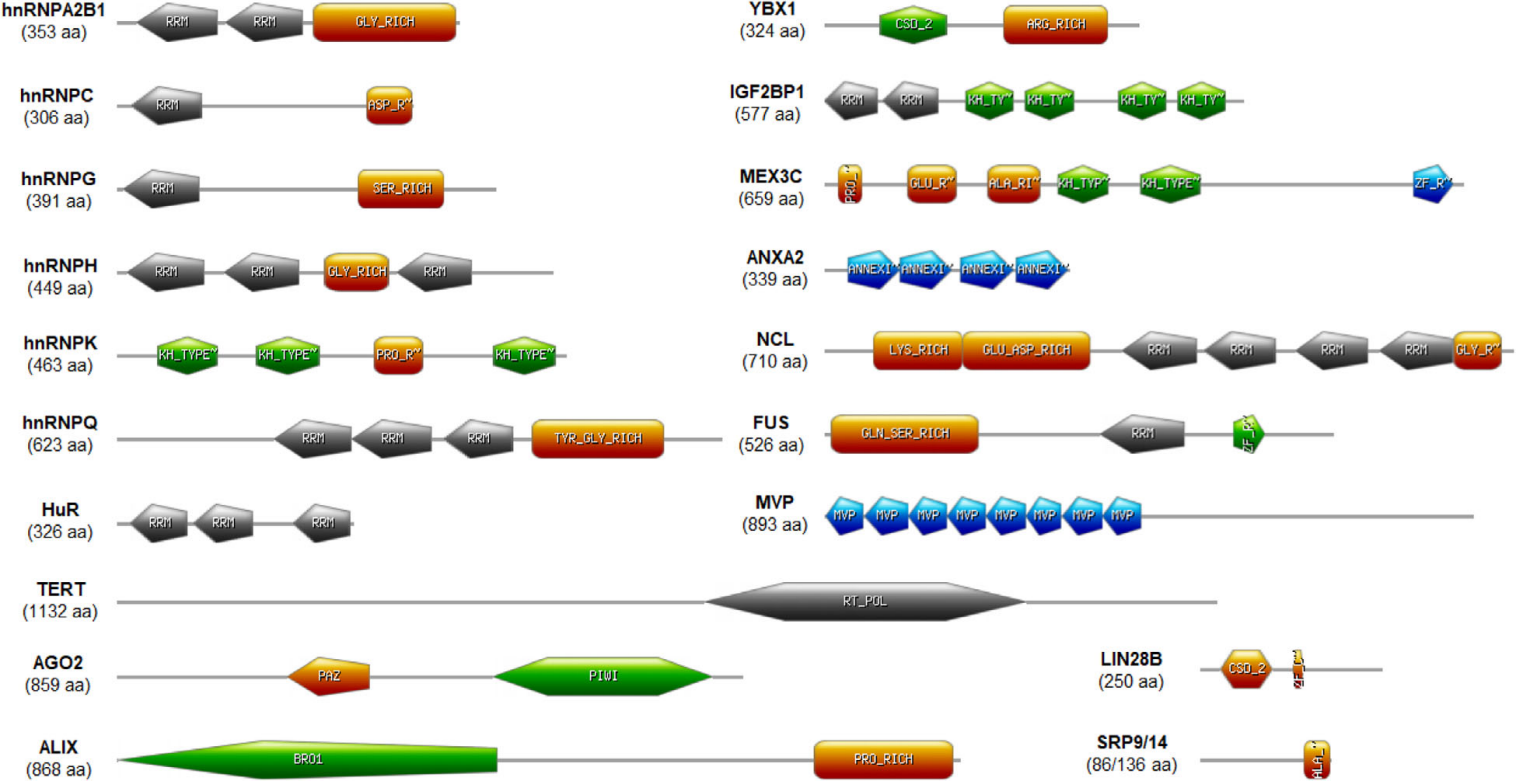
RBPs identified in association with EVs and principal protein domain features. Secondary structures were recognized using primary sequence analysis by ProScan. RRM (RNA recognition motif), GLY_RICH (Glycine‐rich region profile) KH_TY (hnTNP K Homology domain type 1), ZF_RANBP2_2 (Zinc finger RanBP2 type profile), CSD_2 (Cold‐shock domain profile), ZF_CCHC (Zinc finger CCHC‐type profile), ANNEXIN_2 (Annexin repeat profile), BRO1 (BRO1 domain profile), PAZ (PAZ domain profile), PIWI (Piwi domain profile), RT_POL (Reverse transcriptase catalytic domain profile). QKI protein domains were not detected in ProScan

Irrespective of the methodologies used to isolate EVs, we highlight the protein domain features of RBPs detected into EVs (Figure 1), the previous knowledge on their association with human diseases, the EV‐RNA consensus sequences responsible for the interaction between the RBPs, and known small molecules inhibitors of RBP activity and interactions.

Here, we describe members of the hnRNP family (hnRNPA2B1, hnRNPC1, hnRNPG, hnRNPH1, hnRNPK, and hnRNPQ), as well as YBX1, HuR, AGO2, IGF2BP1, MEX3C, ANXA2, ALIX, NCL, FUS, MVP, LIN28, SRP9/14, QKI, and TERT RNA‐binding proteins.

## 2 THE hnRNP FAMILY

Heterogeneous nuclear ribonucleoproteins (hnRNPs) represent a large family of 20 major and minor proteins in humans with differential RNA‐binding capacities (Dreyfuss, 1993). hnRNPs are characterized by a typical structure of four unique RNA‐binding domains (RBDs): the RNA recognition motif (RRM), the quasi‐RRM (qRRM), a glycine‐rich domain constituting an RGG box, and a KH domain. The RRM is the most common, while the glycine‐, proline‐, or acid‐rich domains seem auxiliary structures responsible for homologous or heterologous interactions with other hnRNPs (Dreyfuss et al., 2002). As also highlighted in Figure 1, all RBDs are not always present, and the RNA‐recognition specificity is mediated by flexible protein conformations and post‐translational modifications that finely tune the RNA‐protein interactions (Geuens et al., 2016).

Six members of this RBP family emerged in association with EVs: hnRNPA2B1, hnRNPC1, hnRNPG, hnRNPH1, hnRNPK, and hnRNPQ. These RBPs are ubiquitously expressed factors involved to a different extent in the maturation, transport, stability, and translational efficiency of mRNAs and the metabolism of miRNAs. Altered expression of these proteins has been reported in several cancer types and in associations with clinical outcomes. Table 1 summarizes the relevant intracellular functions and the specific involvement in disease described so far for these proteins. In the text, we highlight the recent studies implicating individual hnRNP members' activity in association with extracellular vesicles.

**Table:** 

Member	Functions	Involvement in disease
hnRNPA2B1	‐Alternative splicing (Martinez‐Contreras et al., 2007) ‐mRNA transport (Han et al., 2010) ‐Pri‐miRNA maturation (Alarcón et al., 2015) ‐Telomere maintenance (Moran‐Jones et al., 2019) ‐Innate immunity against DNA viruses (Wang et al., 2019)	‐ Recognized as autoantigen in RA and SLE (Wu et al., 2018) ‐ Prion‐like properties in ALS, FTD, AD (Harrison & Shorter, 2017) ‐ Overexpressed in lung cancer, pancreatic adenocarcinoma, breast cancer (Barceló et al., 2014; Hung et al., 2017; Klinge et al., 2019)
hnRNPC1	‐ Formation of RNP complex with pre‐mRNA (Rech et al., 1995) ‐ Splicing regulation (König et al., 2010; Venables et al., 2008) ‐ Transport of mRNA and snRNAs (Mccloskey et al., 2012) ‐ Translation enhancer (Cieniková et al., 2015)	‐ hnRNPC1 targets pri‐miR‐21 in glioblastoma multiforme, controlling its metastatic potential (Park et al., 2012) ‐ Negative prognostic factor in advanced gastric cancer (Huang et al., 2016)
hnRNPG (RBMX)	‐ Alternative splicing (Heinrich et al., 2009) ‐ Chromosome integrity and repair (Matsunaga et al., 2012) ‐ Chromosome segregation (Cho et al., 2018) ‐ Genome stability (Munschauer et al., 2018)	‐ *RBMX* somatic mutations and altered expression levels associate with lung, endometrial and other solid tumours (Elliott et al., 2019) ‐ Overexpression is associated to poor prognosis in oral squamous cell carcinoma (Guo et al., 2020)
hnRNPH1	‐ Alternative splicing (Alkan et al., 2006) ‐ DNA replication (Barrack, 1987) ‐ RNA transcription (Barrack, 1987) ‐ Inhibition of cell differentiation (Liu et al., 2001)	‐ Overexpression associates with poorly differentiated solid tumours (head, neck, hepatocellular, pancreatic, colon, laryngeal, oesophageal, and prostate) (Sun et al., 2016) ‐ hnRNPH1 mRNA in exosomes was suggested as a biomarker for HCC (Xu et al., 2018) and advanced prostate cancer (Yang et al., 2016)
hnRNPK	‐ Regulation of mRNA stability and translation (Xu et al., 2019)	‐ hnRNPK targets lncRNAs involved in cancer progression: SCAT7, pancEts (Ali et al., 2018) and SLINKY (Gong et al., 2017) ‐ Frequently overexpressed and associated with poor prognosis in various malignancies (Chen et al., 2008; Hong et al., 2014) ‐ Downregulation of hnRNPK limits tumour growth (Tang et al., 2014)
hnRNPQ (SYNCRIP)	‐ Alternative splicing (Chen et al., 2008) ‐ mRNA stability (Williams et al., 2016) ‐ RNA transport (Rossoll et al., 2002)	‐ SYNCRIP, overexpression correlates with late‐onset SMA III, as it interacts with SMA‐related mutant SMN1 (Helmken et al., 2003; Yoo et al., 2009) ‐ Downregulation associated with decreased proliferation and chemoresistance in colon cancer (Lai et al., 2017)

**TABLE 1:** Technical and biological indications about RBPs identified in EVs

Protein	EV isolation method	PTMs in EVs	Main interactors	RNA motifs	Biological indication in EVs	Inhibitors
hnRNPA2B1	Differential ultracentrifugation Polymer precipitation	Sumoylation O‐GlcNacylation	PTBP1, HNRNPL, HNRNPH1, SRSF1, TRA2B, HNRNPC, HNRNPA1, HNRNPF, HNRNPK, SRSF3	A/G‐rich motifs 5′‐AGG 5′‐UAG 5′‐GGAG	RBP and RNA substrates	Epirubicin MO‐460 Compound 2155‐14 Several planar aromatic compounds
hnRNPC1	Differential ultracentrifugation	ND	HNRNPA1, HNRNPA2B1, ALYREF, HNRNPL, HNRNPM, CDC5L, HNRNPK, ELAVL1, SRSF1, HNRNPH1	AU‐rich elements (AREs)	RBP and RNA substrates	ND
hnRNPG	Differential ultracentrifugation	ND	HNRNPK, CDC5L, HNRNPH1, TR2B, HNRNPA1, HNRNPL, PTBP1, HNRNPC, SRSF3, HNRNPR	5’‐CC[A/C]‐rich	RBP and RNA substrates	ND
hnRNPH1	Differential ultracentrifugation	ND	HNRNPA1, HNRNPK, HNRNPA2B1, HNRNPM, HNRNPA0, SRSF1, HNRNPF, HNRNPC, TRA2B	5′‐GGGA	RBP and RNA substrates	Compound 2155‐14
hnRNPK	Differential ultracentrifugation Polymer precipitation	ND	HNRNPM, HNRNPA1, HNRNPH1, PTBP1, RBMX, HNRNPL, HNRNPF, ELAVL1, HNRNPA2B1, HNRNPC	5′‐UC_3–4_(U/A)_2_	RBPs and RNA substrates	Nujiangexathone Quinoline derivatives Psammaplysene A
hnRNPQ (SYNCRIP)	Differential ultracentrifugation Polymer precipitation Ultrafiltration	ND	APOBEC1, A1CF, PAPC1, PAIP1, CSDE1, HNRNPD, HNRNPR, SMN, HABP4, DHX9, HNRNPU, IGF2BP1, YBX1, ELAVL1	5′‐AYAAYY 5′‐UAUYRR 5′‐GGCU	RBP and RNA substrates	ND
YBX1	Differential ultracentrifugation Polymer precipitation Ultrafiltration Density gradient ultracentrifugation	Dephosphorylation (T271)	SRNPD1,HNRNP, SF3A2, HNRNPA2B1, HSPA8, SYNCRIP, LSM3, HNRNPK, ZCRB1, HNRNPA1	5′‐ACCAGCCU 5′‐CAGUGAGC 5′‐UAAUCCCA	RBP and RNA substrates	RUS0207‐A006, RUS0202‐G005 K0395‐B007 Fisetin HSc025
HuR	Differential ultracentrifugation	Ubiquitination	PTBP1, hnRNPK, hnRNPC, CDC5L, hnRNPL, hnRNPA2B1, SRSF3, TRA2B, hnRNPA1, SRSF1	AU‐rich elements (AREs)	RBP and RNA substrates	Among others, DHTS MS‐444
AGO2	Differential ultracentrifugation Density gradient ultracentrifugation	Phosphorylation (S387)	DICER1, TNRC6A, TARBP2,TNRC6C, TNRC6B, XPO5, DROSHA, MOV10, DHX9, PRKRA	G‐rich sequences 5′‐GCACUU	RBP and RNA substrates	4c and 4e BCI‐137 acriflavine aurintricarboxylic acid, suramin oxidopamin
IGF2BP1	Differential ultracentrifugation Density gradient ultracentrifugation	ND	IGF2BP3, SYNCRIP, YBX1, HNRNPU, DHX9, MAPK4, CSDE1, ELAVL4, IGF2, LIN28B	5′‐GGACU 5′‐ACACCC 5′‐AAGCACCCGUU 5′‐GG(m6A)C	RBP and RNA substrates	BTNYB
MEX3C	Differential ultracentrifugation Density gradient ultracentrifugation	ND	RBBP6, FBXW, 2DZIP, 3SPSB, 2LRRC41, KLHL3, MGRN1, SPSB1, TRIM4, ARIH2	5′‐CAGAGUUUAG	RBP and RNA substrates	Triptolide
ANXA2	Differential ultracentrifugation	Phosphorylation (T23)	S100A10, S100A4, PLG, DYSF, PLAT, RPS6KA1, TRIM72, ANXA1, SDCBP, MAPK1	5’‐AA(C/G)(A/U)G	RBP and RNA substrates	1,2,4‐triazole analogues G‐Rg5 G‐Rk1
ALIX	Differential ultracentrifugation	Palmitoylation	CHMP4B, TSG101, CHMP4A, CHMP4C, VPS4B, VPS4A, VPS28, CHMP6, CHMP2A, CHMP5	ND	RBP and RNA substrates	ND
NCL	Polymer precipitation	ND	NPM1, FBL, WDR43, NSUS2, NOP56, NOP58, WDR46, RPS6, HSP90AA1, WDR75	5'‐UUAGGG 5'‐UCCCGA	RBP and RNA substrates	Oridonin Curcumol
FUS	Differential ultracentrifugation	ND	CDC5L, hnRNPD, TNP01, SRSF10, hnRNPA3, SF3A2, SRF2, hnRNPA1, hnRNPC, PTBP1	ND	RBP	ND
MVP	Immunobeads Differential ultracentrifugation Density gradient ultracentrifugation Polymer precipitation Ultrafiltration	ND	TEP1, PTEN, MAPK1, PTPN11, SRC, APEX1 CDC5L, PARP4, TEP1, PARP9, C19ORF48, VWF, PRPF19, PARP10, VIT, ABCC1	Aspecific, RNA‐mediated	Mostly RBP	Silvestrol
LIN28	Differential ultracentrifugation Ultrafiltration	ND	EIF3S2, NCL, DHX9, TUT4, ZCCHC6, TRIM71, DROSHA, DICER1, MYCN, HMGA2, SOX2, IGF2BP1, ZCCHC11, DIS3L2 POU5F1, SOX2, NANOG, SMAD2, SMAD4, KLF4, SALL4, STAT3, PRDM14, ZSCAN10	5’‐GGAGG/GGAGA	RNA substrates	Among others, N‐methyl‐N‐[3‐(3‐methyl[1,2,4]triazolo[4,3‐b]pyridazin‐6‐yl) phenyl]acetamide 6‐hydroxy‐dl‐DOPA SB/ZW/0065 TPEN (LI38)
SRP9/14	Differential ultracentrifugation Polymer precipitation	ND	SRP19, SRP54, SRP68, SRP72, RPL30 with SRP14: RPS17, RPS25, RPS27, RPL27 with SRP9: RPL23A, RPL21, RPL15, RPL23	ND	RBP and RNA substrates	*Scutellaria barbata* polysaccharides (SBPS)
QKI	Differential ultracentrifugation	ND	CARM1, FUBP3, HNRNPK, HNRNPLL, NABP1, PCBP1, PRKAB2, PTBP1, RBFOX1, RBFOX2, RBM11, RBPMS, SNRPA, TIAL1, MED25, MED15, MED14, MED4, MED24, MED12, MED17, MED27, MED1, MED16	5’‐NACUAAY‐N(1‐20)‐UAAY	RNA substrates	ND
TERT	Differential ultracentrifugation Polymer precipitation	ND	DKC1, SMARCA4, PINX1, MYC, XRCC5, XRCC6, PIF1, NHP2, AKT1, SMG6	ND	RBP‐encoding mRNA	GRN163L

EV isolation methods, post‐translational modifications (PTMs), main interactors (by STRING Network Database; https://string-db.org), RNA motifs, biological indication in EVs, and targeting inhibitors are reported. ND: Not yet detected.

Recently, **hnRNPA2B1** emerged as a key player in the sorting of specific miRNAs into exosomes and microvesicles (MVs). The protein was found responsible for direct interaction with a GGAG motif present in the 3′‐end of miRNAs (i.e., miR‐198) enriched in vesicles. Notably, the relevant activity of hnRNPA2B1 was associated with its sumoylation status (Villarroya‐Beltri et al., 2013). Another study showed that the entrapment of hnRNPA2B1‐bound miRNAs into epithelial cell‐derived MVs is mediated by the membranous protein caveolin‐1, which interacted with hnRNPA2B1 upon phosphorylation (Lee et al., 2019). The complex formation induced an O‐GlcNacylation in hnRNPA2B1, essential for the sorting of AGG and UAG motifs‐containing miR‐17 and ‐93 into MVs, guiding hnRNPA2B1 towards MVs rather than exosomes.

On the other hand, hnRNPA2B1 was recently identified as an inhibitor of the exosomal sorting of miR‐503 in endothelial cells (Pérez‐Boza et al., 2020). This group determined that epirubicin increases the exosomal export of miR‐503 in endothelial cells by disrupting the interaction between hnRNPA2B1 and the miRNA, probably mediated by ANXA2 (below described).

Other studies instead highlighted the hnRNPA2B1‐dependent accumulation of long non‐coding RNAs (lncRNAs) within exosomes. Lei et al., (2018) demonstrated that lncRNA H19 is secreted through hnRNPA2B1‐mediated packaging into exosomes, promoting resistance to gefitinib (an EGFR‐tyrosine kinase inhibitor) in NSCLC cells. H19 was significantly reduced in the presence of RNase and Triton X‐100, demonstrating its encapsulation in intact vesicles. The authors found a GGAG motif at the 5’‐end region of H19, which, when mutated, impaired the hnRNPA2B1‐binding ability. hnRNPA2B1 overexpression induced an increase of exosomal H19, while its knockdown decreased H19 expression in NSCLC cells. Han et al., (2020) showed a role for the exosomal AFAP1‐AS1 lncRNA in acquired trastuzumab resistance. hnRNPA2B1 was found crucial for the AFAP1‐AS1 loading into exosomes. This study corroborated the work of Zheng et al., (2019), who demonstrated an increased encapsulation of AGAP2 antisense RNA 1 mediated by hnRNPA2B1 in SKBR‐3R and BT474R cells in association with trastuzumab resistance. Chen et al., (2020) described a direct interaction between hnRNPA2B1 and lncRNA LNMAT2 in bladder cancer (BCa) cells, promoting lymphatic metastasis in a VEGFC–independent manner. They identified the specific exo‐motif GGAG as a determinant for direct interaction with hnRNPA2B1, further confirmed by site‐directed mutagenesis experiments. These data suggest a pivotal role of hnRNPA2B1 in the selection of cancer‐associated miRNAs and lncRNAs, emphasizing the function of this RBP in connection with the vesicular trafficking.

Given the multiple roles of hnRNPA2B1 in disease, some screening campaigns identified small molecules proposed to interact with the RNA‐binding activity of the protein. MO‐460, an analogue of the natural product (R)‐(‐)moracin‐O, suppresses the accumulation in Hep3B cells of HIF‐1α (Hypoxia‐inducible factor‐1α), involved in tumour cell adaptation to hypoxic conditions. MO‐460 binds to the C‐terminal glycine‐rich domain of hnRNPA2B1, inhibiting its subsequent binding to the 3'‐untranslated region of HIF‐1α mRNA (Soung et al., 2019). Fang et al. identified daunorubicin, pyrvinium, and pararosaniline as active planar aromatic moieties able to disrupt the RNA‐dependent recruitment of the ALS‐associated TDP‐34, FUS, and hnRNPA2B1 into stress granules, reducing progression to pathological protein aggregates (Fang et al., 2019).

Balaguer et al. identified **hnRNPC1** as a protein influencing the exosome loading of miR‐30d, a transcript involved in embryonic adhesion processes (Vilella et al., 2015). Co‐localization studies of hnRNPC1 and CD63 indicated exosomes carrying hnRNPC1, whose level associated with miR‐30d biogenesis in epithelial‐like cells. Interestingly, co‐culture assays of miR‐30d KO and silenced hnRNPC1 revealed a decrease in embryo‐miR‐30d acquisition during the adhesion and invasion stages. Mechanistically, a putative direct binding of hnRNPC1 to miR‐30d was proposed at the intracellular level before the loading into the inner cavity of exosomes (Balaguer et al., 2018).

The chromosome‐linked RNA‐Binding Motif X (**RBMX**), also known as hnRNPG, as an ARTS‐1 (aminopeptidase regulator of TNFR1 shedding)‐associated protein, is involved in two mechanisms of TNFR1 secretion: the constitutive release through exosome‐like vesicles and the IL‐1β‐mediated inducible proteolytic cleavage of TNFR1 ectodomains. The RBMX RNAi reduces, in fact, TNFR1 release through both mechanisms (Adamik et al., 2008). An altered abundance of RBMX was shown in endothelial cell‐derived exosomes upon cellular stress, and the presence of the protein was also observed by proteomic analysis in exosomes isolated from ovarian cancer cells (de Jong et al., 2012; Liang et al., 2013).

More recently, Ahadi et al. investigated the presence of miRNA seed regions and specific RBP binding sites on lncRNAs in exosomes derived from four prostate cancer cell lines (VCaP, LNCaP, DU145, PC3) and healthy prostatic cells (Ahadi et al., 2016). They found hundreds of lncRNAs enriched in cancer exosomes and 38 predicted RBPs with putative binding sites. Among them, RBMX, SFRS1, and ELAVL1 resulted in having the highest number of predicted binding sites. Through their algorithm, the group identified 126 distinct six‐base motifs exclusively enriched in the four prostate cancer cell lines: RBMX gave positive matching in VCaP, PC3, and LNCaP cell lines.

Recently, the mRNA of **hnRNPH1** was detected in exosomes isolated from the serum of hepatocellular carcinoma (HCC), chronic hepatitis B (CHB), and liver cirrhosis (LC) patients, resulting to significantly higher levels in patients compared to control groups. Also, high exosomal hnRNPH1 mRNA levels were associated with worse overall survival (OS) in HCC patients (Xu et al., 2018). Together with other proteins, Statello et al. identified hnRNPH1 in their exosome extracts capable of interacting with exo‐RNAs and cell mRNAs. Preliminary experiments revealed an unbalanced trend in the relative abundance of total RNA at the intracellular level in human epithelial cells and in derivative exosomes. For this inverse correlation with hnRNPH1 protein levels, the authors hypothesized a possible inhibitory pathway sustained by hnRNPH1 to retain cytoplasmic RNAs at the intracellular level.

These observations exist in parallel with data reported by Datta et al., who identified hnRNPH1 as a partial mediator of the RAS‐dependent Manumycin‐A (MA) suppression of exosome biogenesis and release (Datta et al., 2017). In this study, the natural microbial metabolite MA inhibited exosome release in castration‐resistant prostate cancer (CRPC) cells through the reduction of RAF and ERK and also the attenuation of ESCRT‐0, ALIX, and Rab27a proteins. The authors found hnRNPH1 as an intermediate factor inhibited by MA and responsible for the decreased levels of Alix and Rab27a transcripts. The silencing of hnRNPH1 through shRNA confirmed the decreased release of EVs along with a reduced expression of cellular Ras, pERK, Alix, Rab27a, and CD9 proteins. Of note, the hnRNPH1 mRNA in blood exosomes was suggested as a biomarker for HCC (Xu et al., 2018) or advanced prostate cancer (Yang et al., 2016).

The compound 2155‐14, recently found as an inducer of autophagy in melanoma cells, was identified as a direct interactor of the RNA helicase DDX1, as well as of hnRNPH1, H2, and hnRNPA2/B1 (Palrasu et al., 2019). Given the demonstrated effect of this drug on ER‐stress marker RNAs regulated by these RBPs, it could be plausible a potential investigation of this compound as a function of hnRNP modulation and RNA sorting into EVs.

Gao et al. indicated a role of **hnRNPK** in exosomes mediated transfer of lncRNA 91H in the context of colorectal cancer (CRC) progression. The overexpression of lncRNA 91H was also observed in exosomes from HCT‐8, HCT‐116 cell lines, and in patients’ serum. Mass spectrometry analysis revealed a physical interaction between hnRNPK and lncRNA 91, further confirmed by RNA interference (Gao et al., 2018). Statello et al. also found this protein in their biochemical assays using RNA extracts from cells and exosomes (Statello et al., 2018). Recently, Leidal et al. identified the LC3‐dependent EV loading and secretion (LDELS) of RBPs, such as hnRNPK and SAFB. This process employed a pool of LC3‐II (endogenous LC3) located at the MVB limiting membrane to directly capture RBPs and package them into ILV for the subsequent release as EVs *via* MVB fusion with the plasma membrane (Leidal et al., 2020).

In the context of small molecules, the natural compound nujiangexathone was found to repress tumour growth by targeting hnRNPK (Zhang et al., 2016). Moreover, Shu et al. identified the quinoline derivatives as able to disrupt hnRNPK binding to *c‐MYC* gene promoter and a promising approach for cancer therapy (Shu et al., 2019). Psammaplysene A (PA), a neuroprotective drug, was found to interact with hnRNPK, suggesting a possible role for HNRNPK in neuroprotection (Boccitto et al., 2017). A biochemical understanding of the interaction between hnRNPK and its protein partners has been proposed as necessary to establish hnRNPK as a potential therapeutic target in different cellular contexts (Wang et al., 2020).

Synaptotagmin‐binding Cytoplasmic RNA Interacting Protein (**SYNCRIP**, also known as hnRNPQ or NSAP1) has been described as a secreted factor in extracellular vesicles. The presence of SYNCRIP in hepatocellular exosomes was linked to the control of specific miRNAs (Santangelo et al., 2016), such as miR‐3470a and miR‐194‐2‐3p, previously associated with liver regeneration and hepatocellular carcinoma, respectively (Schug et al., 2013; Zhao et al., 2015). Additionally, Santangelo et al. identified an exosome‐enriched GGCU consensus motif responsible for the packaging of miRNAs into the vesicles. Remarkably, the SYNCRIP‐dependent RNA cargo did not overlap with the hnRNPA2B1‐dependent one, as demonstrated from the analysis of miRnome upon RBP silencing (Santangelo et al., 2016).

Summarizing, these data indicate that the intracellular balance of RBP isoforms can be responsible for a selective entrapment of RNA species into EVs, with a potential impact of post‐transcriptional control in the physiological tissue homeostasis.

## 3 YBX1

The Y‐box binding protein 1 (YBX1) is a RNA‐binding protein composed of an A/P rich domain (alanine/proline‐rich domain), a cold shock domain (CSD), and a long C‐terminal domain with alternating positively and negatively charged amino acids (Eliseeva et al., 2011). The human protein is composed of 324 residues (Figure 1) that can recognize the Y box motif (5'‐CTGATTGGCCAA) in the promoter of the major histocompatibility complex II (*HLA‐DRα*gene), the single‐stranded motif GGGG, as well as either single‐ or double‐stranded forms of the motifs CACC and CATC. The YBX1 A/P domain interacts with SRp30c, p53, and cyclin D1, whereas it's CSD interacts with AKT kinase and E3 ubiquitin ligase. The CTD is involved in protein homodimerization. YBX1 directly interacts with proliferating cell nuclear antigen (PCNA), mutS homolog 2 (MSH2), X‐ray repair cross complementing 5 (XRCC5), and DNA ligase III, clearly indicating its involvement in DNA repair (Eliseeva et al., 2011; Gaudreault, 2004). YBX1 is subjected to post‐translational modifications, such as phosphorylation, ubiquitination, and acetylation, that tune the presence of the YBX1 in mRNPs and polysomes to regulate the translation of many genes involved in cancer (Suresh et al., 2018).

The YBX1‐regulated non‐coding transcriptome includes a large number of RNA classes, such as spliceosomal RNAs, lncRNAs, Y RNAs, tRNA‐derived small RNAs, miRNAs, tiny transcription‐initiation RNAs, promoter‐associated short RNAs, termini‐associated short RNAs (TASRs), antisense TASRs, 3ʹUTR‐derived RNAs, and tRNA‐derived short RNAs (tsRNAs and tRFs) (Lee et al., 2009; Prensner & Chinnaiyan, 2011).

YBX1 can co‐localize with cytoplasmic P‐bodies containing members of the RISC complex, including GW182, which can be found in exosomes (Gallois‐Montbrun et al., 2007; Goodier et al., 2007). Interestingly, YBX1 can be found in exosomes secreted by normal and malignant cells as well, like B cells, hepatocytes, prostate, colorectal, and ovarian cancer cells (Suresh et al., 2018). There is biochemical evidence that YBX1 is secreted in a form that resists trypsin in the absence, but not in the presence, of non‐ionic detergent (Triton X‐100), consistently with a location in vesicles, probably exosomes (Frye et al., 2009; Rauen et al., 2009). There is evidence that YBX1 may undergo dephosphorylation at T271 to reach exosomes as an RNP form (Kossinova et al., 2017). A very recent study employed ssODN‐library to target exosomes secreted by VCaP and LNCaP prostate cancer cell lines. Using the adaptive dynamic artificial polyligand targeting (ADAPT), the authors were able to differentiate exosomes from cancer cell subtypes. Specifically, using an easily detectable sequence as a probe for target capturing, the authors identified proteins known to be involved in the formation of the endosomal sorting complexes required for transport (ESCRT) machinery. In these settings, YBX1 was identified as part of this interactome, specifically in complex with ubiquitinylated TSG101 (Hornung et al., 2020).

Shurtleff et al. demonstrated that YBX1 is required for miR‐223 packaging into exosomes released from HEK293T cells. In the absence of statistically significant primary sequence motifs for miRNAs in those vesicles, the authors suggested that sorting mechanisms could be based on secondary RNA structures that YBX1 could escort into exosomes (Shurtleff et al., 2016). In this line, Yanshina et al. evidenced recurrent hairpins in the 3’‐UTR of exosomal transcripts recognized by YBX1 (Yanshina et al., 2018). Subsequent analyses, aimed at defining the YBX1‐dependent exosomal RNA composition, revealed that YBX1 is required to sort other small ncRNAs such as tRNAs, YRNAs, and vault RNAs. Indeed, through TGIRT‐seq on YBX1‐null exosomes from HEK293T cells, Shurtleff et al. observed a proportional reduction of these RNAs in YBX‐1 null exosomes, while the matching YBX1‐null cells were displaying an increase in the corresponding proportion of reads. These data suggested that a YBX1‐mediated blocking of cellular export via the exosome pathway resulted in the accumulation of these transcripts at the intracellular level (Shurtleff et al., 2017).

A recent study by Lin et al. shows that miR‐133 is sorted into hypoxia/reoxygenation (H/R)‐induced human endothelial progenitor cell (EPC)‐derived exosomes *via* YBX1, increasing fibroblast angiogenesis and mesenchymal‐endothelial transition (MEndoT) (Lin et al., 2019). Another work highlights the interplay of YBX1 and NSUN2 proteins in recognizing exosomal RNA through the binding to ACCAGCCU, CAGUGAGC, and UAAUCCCA motifs in the HEK293 S100 extract. Interestingly, these proteins appeared in exosomes together with mRNAs containing these motifs and the degree of enrichment in exosomes correlated with the relative position of the motifs in the mRNA primary structure (Kossinova et al., 2017).

The high expression level of YBX1 is a poor prognostic marker in patients with breast cancer (Habibi et al., 2008), ovarian cancer (Kamura et al., 1999), prostate cancer (Giménez‐Bonafé et al., 2004), and other human malignancies (Kosnopfel et al., 2014). Therefore, YBX1 is an attractive target to develop novel therapies (Kosnopfel et al., 2014). A drug screening campaign carried by Trevarton et al. identified RUS0207‐A006, RUS0202‐G005, and JK0395‐B007 as small molecules interfering with the transcriptional functions of YBX1 (Trevarton et al., 2019). By using the phenotypic Epithelial‐to‐Mesenchymal Transition (EMT) readout in prostate cancer (PCa) models, Khan et al. identified the fisetin compound as a drug to inhibit YBX1 activity (Khan et al., 2014). In another disease context, Higashi et al. identified the small molecule HSc025 as a direct ligand of YBX1 able to disrupt the interaction with PABP, resulting in accelerated nuclear translocation of YBX1 with an improvement from liver injury and hepatic fibrosis in mice (Higashi et al., 2011).

## 4 HuR (ELAVL1)

Human antigen R (HuR or ELAVL1) is a member of the embryonic lethal abnormal vision (ELAV) family of RNA‐binding proteins (Simone & Keene, 2013). HuR is one of the most widely studied RBPs involved in the post‐transcriptional control of a plethora of RNA targets in different cellular contexts. From the nucleus to the cytoplasm, it regulates the splicing, transport, stability, and translation of thousands of coding and noncoding RNAs bearing AU‐rich elements (AREs) (Zucal et al., 2015). These sequences, mainly found in the 3’‐untranslated region, mediate the affinity of the protein and are present in genes involved in inflammation, cell division, angiogenesis, senescence, apoptosis, immune, and hypoxia response. Interestingly, the interplay among different RNA species highlighted the involvement of HuR in regulating critical miRNAs involved in development (miR‐126), cell homeostasis (miR‐126, miR‐92a), and pathological angiogenesis (miR‐200b, miR‐132), as reviewed by Chang and Hla (Chang & Hla, 2014).

Structurally, HuR contains two RRMs at the N‐terminal region, determining the recognition of the RNA, while a third RRM (RRM3) in the C‐terminal region seems implicated to poly‐A recognition and protein oligomerization (Wang et al., 2013). In cancer, the overexpression of HuR was directly involved in the balance of transcripts that increase cell proliferation and survival. Consistently, the upregulation of HuR, along with its cytoplasmic re‐localization, is associated with disease progression and poor prognosis in patients diagnosed with several types of solid tumours, including breast, colon, ovarian, prostate, pancreatic, and oral cancer (Schultz et al., 2020).

A recent study describing the tumour microenvironment of carcinoma‐associated fibroblasts (CAFs) demonstrated an association between CAFs oxaliplatin resistance and the horizontal transfer of CCAL (colorectal cancer‐associated lncRNA) lncRNA from neighbouring cancer cells (Deng et al., 2020). In this study, Deng et al. showed that this lncRNA, shuttled by colon cancer‐derived extracellular vesicles, directly interacts with HuR in recipient fibroblasts leading to an increase in β‐catenin mRNA and protein levels, triggering survival and a chemoresistant phenotype. A variety of lncRNAs, in turn substrate for several miRNAs, was also identified in prostate cancer exosomes. These lncRNAs displayed an over‐representation of *cis‐elements* inferring the activity of HuR and RBMX RBPs (Ahadi et al., 2016).

Recently, Mukherjee et al. demonstrated that HuR accelerates an export of miRNAs inside EVs (Mukherjee et al., 2016). In this work, the authors show a counter‐interplay of HuR and AGO2 for the binding to miRNAs, balanced by a ubiquitination step of HuR triggered by cellular stress. This PTM was associated with an HuR‐unloaded RNA and a consequent association to MVBs in human hepatocytes. Under conditions of cellular stress, such as starvation, the HuR‐dependent extracellular export of miR‐122 was observed along with CD63‐positive EVs. Interestingly, the loss of blood‐circulating miR‐122 was reported in mice starved for 12 h.

The exploratory approach conducted by Shi et al., who exploited the exosomal RNA in pull‐down assays, revealed that miR‐1246, previously found significantly upregulated in the serum of gastric cancer patients, is packaged into exosomes with HuR involvement, suggesting a rationale to detect miR‐1246 as a potential biomarker for the disease (Shi et al., 2020).

As recently reviewed by Schultz et al., various strategies have been proposed to target HuR, including the inhibition of the shuttling from the nucleus to the cytoplasm, the interference during the RNA recognition step, and the reduction of protein expression levels (Schultz et al., 2020). In the context of small molecules, besides the small molecule inhibitors recently reviewed (D'agostino et al., 2019), we highlight novel indole derivatives tested in models of retinal endothelial cells (Platania et al., 2020), the suramin tested in oral cancer cells (Kakuguchi et al., 2018), the azaphilone‐9 tested in biochemical settings (Kaur et al., 2017), and tanshinone mimics tested in different cancer cells (Manzoni et al., 2018) using specific RNA targets as a readout of HuR activity. Regarding the small molecules with a reported follow‐up testing, we highlight the MS‐444 compound in adenomatous polyposis (Lang et al., 2017) and the DHTS, previously identified to inhibit HuR:RNA interaction (D'agostino et al., 2015), very recently found as able to reduce autoimmune neuroinflammation in mouse models (Chen et al., 2020).

## 5 AGO2

Argonaute proteins constitute an evolutionarily conserved protein family, including two subclasses: AGO proteins and PIWI proteins. These proteins can bind to small non‐coding RNAs, such as shRNAs, miRNAs, and Piwi‐interacting RNAs (piRNAs), mediating gene‐silencing effects on their RNA targets (Elkayam et al., 2012; Höck & Meister, 2008; Müller et al., 2020). In humans, four AGO proteins (hAGO1 ‐ 4) are highly expressed and share ∼85% of sequence identity with four conserved structural domains: The N‐terminal domain (N), the PIWI/Argonaute/Zwille (PAZ) domain, the MID domain, and the P‐element‐induced wimpy testis (PIWI) domain (Höck & Meister, 2008; Meister, 2013).

In somatic cells, AGO proteins are localized in the cytoplasm and take part in processing bodies (P‐bodies) and stress granules (Höck & Meister, 2008; Meister, 2013). It has been extensively reported that eukaryotic AGOs play a crucial role in transcriptional and post‐transcriptional silencing processes, as components of the RNA‐induced silencing complex (RISC). Small RNAs, such as miRNAs, serve as guide to AGO proteins to recognize partially complementary sequences on mRNA targets, facilitating the downstream silencing effects (Höck & Meister, 2008; Meister, 2013). Moreover, AGO proteins can interact with GW182 protein family (also known as TNRC6 proteins), which recruits AGOs to target RNAs (Meister, 2013). At this point, AGO proteins can coordinate the RNA inducing silencing processes by repressing the target mRNA through RNA degradation or translation inhibition (Höck & Meister, 2008; Müller et al., 2020). AGO proteins have also been found involved in alternative splicing and DNA double‐strand break repair mechanisms (Meister, 2013; Müller et al., 2020) in cancer (Ye et al., 2015) or other diseases like diabetes (Florijn et al., 2019).

AGO2 is the most characterized AGO protein. Through the interaction with the endoribonuclease Dicer1 and TRBP (transactivation response element RNA‐binding) proteins to form the RISC loading complex, it processes precursor miRNAs into mature miRNAs (Macrae et al., 2008).

Given the remarkable enrichment of small‐sized RNA in a typical profile of extracellular vesicle lysates, multiple studies focused on the association between AGO2 activity and the potential sorting of miRNAs. Interestingly, AGO2 was found in complex with miR‐223 in microvesicles released by platelets, further proved to be functional in regulating miR‐223 in recipient endothelial cells (Ye et al., 2015). Gibbins et al. showed that AGO2, together with GW182 protein, localizes to endosomes and MVBs, suggesting that the sorting of miRNAs inside EVs might be mediated by the RISC complex (Gibbings et al., 2009). Later, a study from McKenzie et al. confirmed the association of AGO2 with MVBs in colon cancer cells and demonstrated that the activation of KRAS‐dependent MEK‐ERK signalling was able to hamper the sorting of AGO2 and AGO2‐regulated miRNAs inside extracellular vesicles (Gibbings et al., 2009). AGO2 protein is highly subjected to post‐translational modifications, such as phosphorylation, which influences its cellular localization (Müller et al., 2020). McKenzie et al. observed that the phosphorylation of AGO2 at Serine 387 prevented the secretion of AGO2 and the sorting of specific miRNAs inside vesicles (Mckenzie et al., 2016; Müller et al., 2020). However, other studies pointed out that a form of AGO2 is also highly present in the extracellular space in a vesicle‐free form, bound to circulating miRNAs, even more represented than EVs (Arroyo et al., 2011; Turchinovich et al., 2011; Weaver & Patton, 2020). Indeed, the detection of AGO2 inside vesicles could be dependent on the EV isolation procedure and/or better revealed in certain cell types (Weaver & Patton, 2020). An interesting study by Arroyo et al in 2011 also highlighted how, among a subset of circulating miRNA associated with AGO in human plasma and serum, only a minority was found in vesicles (Arroyo et al., 2011).

Recently, compounds able to bind AGO2 were described. In the hypothesis that an interference with microRNA's seed region could inhibit the recognition of a target mRNA and the cargo mediated by AGO2, Schmidt et al. combined a short nucleotide sequence with a small molecule moiety targeting the AGO2 active site (Schmidt et al., 2013). This strategy evolved a PNA‐based approach to target microRNA‐122 (Schmidt et al., 2013). In another study, Masciarelli et al. employed a structure‐based molecular design approach and identified a cell‐permeable small molecules (BCI‐137) able to compete with AGO2:miRNAs interaction (Masciarelli et al., 2014). This compound mimicked the silencing of AGO2 by lentiviral vector shRNAs in acute promyelocytic leukaemia models favouring granulocytic differentiation of that cells in response to retinoic acid.

## 6 IGF2BP1

The insulin‐like growth factor 2 mRNA‐binding protein 1 (IGF2BP1, alias IMP1, ZBP1, CRD‐BP, VICKZ1) is a member of the insulin‐like growth 2 proteins, a family of RBPs conserved from insects to mammals (Nielsen et al., 1999). IGF2BP1 consists of two RRM domains at the N‐terminus and four hnRNPK homology (KH) domains (Nielsen et al., 2002) (Figure 1). IGF2BP1 mainly localizes in the perinuclear region, with dynamic distribution towards lamellipodia, RNP granules, and microtubular network dependent on KH domains (Farina et al., 2003; Nielsen et al., 2002). The RNA is recognized upon dimerization also in heterodimers with IMP2 and IMP3 members of the IMP family (Chao et al., 2010; Nielsen, 2004). IGF2BP1 is involved in the maintenance of cell polarity as it binds to the zipcode sequence in the 3’UTR of β‐actin mRNA, resulting in transcript translocation to lamellipodia (Farina et al., 2003; Vikesaa et al., 2006). A role of IGF2BP1 was also reported in mRNA stability, as the knockdown of this protein prevents mRNA decay in stress granules during integrated stress response (StöHr et al., 2006). Jønson et al. showed that IGF2BP1 forms specific mRNPs granules unrelated with stress conditions and exploits microtubules to translocate transcripts outside the perinuclear regions (Jønson et al., 2007). IGF2BP1 participates in the autoregulatory translational control of poly‐A‐binding protein as it forms ribonucleic complexes with containing 5’UTRs (Patel, 2005). Interestingly, IGF2BP1 also binds to non‐coding RNAs, such as oncofetal H19, which contains four IGF2BP1‐high affinity recognition sites at the 3’ end (Runge et al., 2000). Electrophoretic mobility shift assays revealed the importance of GGACU and ACACCC motifs in the 54‐nucleotides element zipcode at the 3’UTR of *ACTB* mRNA (Chao et al., 2010; Ross et al., 1997). Farina et al. used RNA selection amplification (SELEX) to identify in *ACTB* 3’UTR an 11‐mer consensus AAGCACCCGUU after eight rounds of selection, recently demonstrated to be IGF2BP1 target‐specific due to the recognition of few amino acids in the variable loops (Biswas et al., 2019; Farina et al., 2003). Enhanced CLIP (eCLIP) experiments allowed the detection of thousands of IGF2BP1 binding sites within RNA targets in human pluripotent stem cells, mainly occurring in 3’UTR of genes related to cell adhesion and extracellular matrix reorganization (Conway et al., 2016).

## 7 I

IGF2BP1 is overexpressed in non‐small cell lung cancer (NSCLC) and associated with poor prognosis (in lung adenocarcinoma) at earlier onset: more than 3‐fold increased IGF2BP1 expression can be detected in 70% of NSCLCs where it positively correlates with a 30% reduction in tumour‐specific 5‐years survival after surgery (Kato et al., 2007; Shi et al., 2017). Rosenfeld et al. reported a synergistic phenomenon between oncogenic KRAS mutation and IGF2BP1 overexpression in murine and human lung carcinoma models: KRASG12V mutant/IGF2BP1 transgenic mice presented an accelerated tumour progression related with *KRAS* mRNA association to IGF2BP1 (Rosenfeld et al., 2019). Additionally, IGF2BP1 overexpression is associated with poor prognosis also in ovarian carcinoma (Köbel et al., 2007) in colon cancer (Dimitriadis et al., 2007), and hepatocellular carcinoma (Gutschner et al., 2014). Unexpectedly, IGF2BP1 has also been correlated with a tumour‐suppressive role in breast cancer (Lapidus et al., 2007) and gallbladder carcinoma (Kessler et al., 2017), suggesting the existence of a cell‐specific post‐transcriptional regulation.

IGF2BP1 was reported as top enriched protein in exosomes derived from murine metastatic pancreatic cell line (Panc02‐H7) compared to exosomes derived from less aggressive cells (Panc02) (Yu et al., 2017). In this study, Panc02‐H7 exosomes enhanced the formation of pre‐metastatic niches in the liver of C57BL/6 mice (Yu et al., 2017).

A biological effect in recipient cells was reported by Nguyen et al., who used EVs derived from mice and human atherogenic macrophages enriched in miR‐146a. The exposure of miR‐146a‐carrying exosomes to naïve macrophages resulted in the inhibition of the migratory pathways through the repression of *IGF2BP1* expression (Nguyen et al., 2018). This observation is consistent with a recent finding on mesenchymal stem cells (MSC)‐derived exosomes and the formation of atherosclerotic plaque. MSC exosome‐associated miR‐let7 inhibited M2 macrophages infiltration and polarization in apoE‐/‐ mice aortic root as a consequence of HMGA2 and IGF2BP1‐mediated mRNA post‐transcriptional repression (Li et al., 2019).

A recent article reported the role of IGF2BP1 in the selection of RNA cargo in metastatic melanoma‐derived EVs. Ghoshal et al. showed, in two different mouse models, that the shRNA‐mediated knockdown of IGF2BP1 altered the total gene expression profile of pro‐metastatic genes in EVs. The authors provided a list of differently enriched RNA motifs between the upregulated (top enriched motif: (A/C)AGGGG) and the downregulated (top enriched motif: AUGACGUA) genes in the transcriptomes which could implicate a direct or indirect sorting mechanism in EVs upon IGF2BP1 depletion (Ghoshal et al., 2019).

An interference with IGF2BP1 cellular function was in the meantime obtained using BTYNB, a recently identified small molecule inhibitor which impaired the binding of the protein to c‐MYC mRNA (Mahapatra et al., 2017).

## 8 MEX3C

MEX‐3 RNA‐binding family member C (MEX3C), also known as RKHD2, BM‐013or RNF194, is a member of an evolutionarily conserved family of four RNA‐binding E3 ubiquitin ligase, known as MEX3 proteins, initially identified in *Caenorhabditis elegans* (Buchet‐Poyau et al., 2007). A study of Li et al. identified isoforms of human MEX3C proteins resulting from alternative splicing or alternative transcription initiation (Li et al., 2016). These proteins are characterized by two heterogeneous K homology (KH) RNA‐binding domains and one carboxy‐terminal RING‐finger domain (Li et al., 2016). Yang et al. identified the motif 5'‐CAGAGUUUAG‐3' as MEX3C‐recognition element in target RNAs (Yang et al., 2017). MEX3C is mostly known to regulate MHC‐I through a post‐transcriptional mechanism (Cano et al., 2012). In particular, a study from Cano et al. in 2012 identified the interaction of MEX3C‐KH domains with the 3'UTR of HLA‐A2 mRNA as responsible for its degradation (Cano et al., 2012). The predominant localization of MEX3C is cytoplasmic, organized in dot‐like structures, when the export receptor CRM1 (also known as exportin1 or Xpo1) does not bind to a leucine‐rich nuclear export signal (NES) causing the shuttling to the cytoplasm (Buchet‐Poyau et al., 2007; Li et al., 2016).

MEX3C1, the longest isoform with 659 amino acid residues, was found overexpressed in cells of the innate immune system and in different types of tumours, such as breast, liver, and bladder cancer, where it displayed oncogenic properties by regulating lipid metabolism (Chao et al., 2019). Other evidence suggested it may play a role in regulating energy balance (Han et al., 2014), stem cell self‐renewal and differentiation, and it contributed to hypertension susceptibility (Guzmán et al., 2006).

A recent study indicated the presence of MEX3C within exosomes isolated by hepatocarcinoma cells and normal hepatocytes (He et al., 2015). Later, the role of MEX3C in the sorting of microRNA inside EVs was reinforced by its colocalization with the endolysosomal adaptor‐related protein complex 2 (AP‐2), a cargo adaptor in clathrin‐mediated endocytosis, and AGO2. Analyzing the exosomes derived by silenced MEX3C or AP‐2 cells, they detected a lower expression of mir‐451, whose downregulation was not ultimately ascribed to the activity of MEX3C alone (Lu et al., 2017). These data suggest the existence of a synergy among RBPs for the selection of miRNAs to be packaged in vesicles.

Li et al. identified the small molecule triptolide as an inhibitor of the enzymatic activity of MEX3C, out of 2027 bioactive compounds screened. This natural compound targeted the UBE2S‐MEX3C interaction leading to inhibition of epithelial‐mesenchymal transition in renal epithelial cells (Li et al., 2019).

## 9 ANNEXIN A2 (ANXA2)

The annexins constitute a family of widely distributed, phospholipid‐binding peripheral membrane proteins characterized by the annexin ‘fold’. This motif, which appears either four or eight times in all annexins, has the form G‐X‐G‐T‐(38)‐(D/E), and allows the annexins to shuttle between water‐soluble and membrane compartments in response to fluctuations in calcium concentration. This property suggests an ability to influence membrane‐related events in response to the cell's homeostasis (Hajjar, 1999). Structurally, the annexins have a variable N‐terminal tail and a highly conserved C‐terminal core structure. The core contains four homologous domains (repeats) (I–IV) (except for AnxA6, which has eight repeats), each consisting of five α‐helices (helices A through E) generating a right‐handed super‐helix (Aukrust et al., 2007; Gerke & Moss, 2002). Generally, the AB‐loop and the DE‐loop form the Ca (2+−) binding sites in the annexins (type II and type III), which differ from the well‐characterized EF‐hand motif (Aukrust et al., 2007; Rosengarth & Luecke, 2004; Rosengarth et al., 2001; Thiel et al., 1991). The human annexin II gene spans approximately 40 kb on the long arm of chromosome 15 (15q21) (Hajjar, 1999; Huebner et al., 1988) and codifies for ANXA2 (also known as p36 or annexin II), which is a 36 kDa calcium‐dependent phospholipid‐binding protein (Hajjar, 1999; Xiu et al., 2016). In addition to Ca^2+^, the core structure of ANXA2 binds different extracellular and intracellular ligands, including heparin (Shao et al., 2006) and F‐actin (Filipenko & Waisman, 2001) and both ligands interact with domain IV (Aukrust et al., 2007; Filipenko & Waisman, 2001; Shao et al., 2006). ANXA2 also binds to specific mRNAs translated on cytoskeleton‐attached polysomes (Vedeler & Hollås, 2000) and therefore it is identified as an RNA‐binding protein (Aukrust et al., 2007). ANXA2 binds to a ∼100 nucleotides region in the 3′‐untranslated regions of its cognate (Hollås et al., 2006). Among transcripts, the c‐*MYC* mRNA (Mickleburgh et al., 2005) contains the consensus sequence 5’‐AA(C/G)(A/U)G, although this stretch alone is not sufficient to ensure binding to ANXA2, which requires an ‘ordered’ RNA structure (Hollås et al., 2006; Vedeler et al., 2012).

ANXA2 is found in the cytoplasm as a monomer or in the cell surface as a heterotetramer consisting of two ANXA2 monomers bridged non‐covalently to an S100A10 dimer (Gerke & Moss, 2002; Woodham et al., 2014). ANXA2 is involved in endocytosis (Emans et al., 1993; Morel & Gruenberg, 2007) and membrane trafficking (Babiychuk & Draeger, 2000). Its role has been also highlighted in cancer, enhancing proliferation, adhesion, migration, invasion, and angiogenesis of different tumour cells (Lokman et al., 2011; Shiozawa et al., 2008; Wang et al., 2012; Xiu et al., 2016). ANXA2 knockdown upregulated p53 and caused cell cycle arrest of non‐small‐cell lung carcinoma cells (Wang et al., 2012). Interestingly, ANXA2 acted as a downstream effector of EGFR signalling in breast cancer cells (Ackland et al., 2003; Grewal & Enrich, 2009), inhibiting the pathway leading to the activation of survival proteins (pAKT, pERK, pSTAT3) (Shetty et al., 2012).

Recently, there is an increasingly recognized role of annexins in influencing the transport and metabolism of coding and non‐coding RNAs (Monastyrskaya, 2018). They can directly or indirectly associate with RNAs and are horizontally transferred between cells through exosomes and microvesicles (Monastyrskaya, 2018). The post‐translational modifications in ANXA2, such as phosphorylation of Tyr23 (Glenney & Tack, 1985), phosphorylation of Ser11 (Jost & Gerke, 1996), and Ser25 (Gould et al., 1986; Johnsson et al., 1986) in the N‐terminal domain, as well as Ser1 acetylation (Johnsson et al., 1986), S‐Glutathionylation of Cys8 (Caplan et al., 2004; Sullivan et al., 2000) and Cys132 (Caplan et al., 2004), determine the interaction with proteins and RNAs. Tyr23 phosphorylation of ANXA2 is required for its Ca^2+^‐dependent association with endosomes and lipid rafts before entering exosomes (Morel & Gruenberg, 2009; Valapala & Vishwanatha, 2011; Vedeler et al., 2012). On the other hand, Tyr phosphorylation could be involved in the regulation of mRNA transport and translation by acting as a negative modulator of ANXA2‐mRNA interaction (Filipenko & Waisman, 2001; Vedeler et al., 2012), although the extent of these interactions is not fully elucidated (Monastyrskaya, 2018).

Hagiwara et al., in 2015, demonstrated that ANXA2 plays a significant role in the loading of miRNAs into EVs (Hagiwara et al., 2015). In this work, they examined a set of miRNAs (miR‐16, miR‐21, miR‐24, miR‐29a, miR‐100, miR‐125, let‐7a, and let‐7b) previously identified in PC3‐derived EVs (Kosaka et al., 2012), and found that their presence in vesicles associated with the levels of intracellular ANXA2 protein. In control experiments overexpressing ANXA2, the protein was found increased in both cells and EVs, with enrichment of miR‐16 in EVs and no significant effects of this transcript in cells, corroborating the evidence that ANXA2 regulates the packaging process of miRNAs into secreted EVs. Interestingly, Ca^2+^ modulated the binding efficiency of ANXA2 to miRNAs and highlighted the relevance of this factor in influencing the loading process of miRNAs in EVs (Hagiwara et al., 2015).

Another study in 2020 reported the interplay between ANXA2 and hnRNPA2B1 as key mediators of the exosomal export of miR‐503 (Pérez‐Boza et al., 2020). The authors demonstrated that, in endothelial cells, the chemotherapeutic drug epirubicin induced the increased exosomal export of miR‐503 by disrupting the interaction between hnRNPA2B1 and miR‐503. They also observed that, upon treatment, hnRNPA2B1 relocates in the nucleus while a fraction of the initial MicroRNA exporting complex (MEC), composed by ANXA2 and miR‐503, is remarkably sorted into exosomes despite the modest variation at intracellular level (Pérez‐Boza et al., 2020).

Other examples underlined of small molecules in blocking the interaction of ANXA2 with other proteins and the cell secretory mechanism. Reddy et al., in 2012, identified substituted 1,2,4‐triazole analogues as inhibitors of the ANXA2–S100A10 protein interaction (Reddy et al., 2012). These molecules resulted able to compete with the binding of the ANXA2 N terminus to S100A10, disrupting protein‐protein interaction (PPI) (Reddy et al., 2012; Woodham et al., 2014). Moreover, it has been shown by Jung et al. that ANXA2 promotes NF‐κB activation by binding to NF‐κB p50 subunit with its N terminal sequences, increasing its transcriptional activity and upregulating the transcription of several target genes downstream of NF‐κB, including interleukin (IL)‐6, which contributes to anti‐apoptotic signalling (Jung et al., 2015). Wang et al., in 2018, demonstrated that two ginsenosides Rg5 (G‐Rg5) and Rk1 (G‐Rk1), with similar structure, directly bound to ANXA2 (Wang et al., 2018), inhibiting its interaction with the NF‐κB p50 subunit and leading to caspase activation and apoptosis. These studies demonstrated that inhibition of ANXA2 provided a new regulatory tool on NF‐κB activity and supported the notion that the inhibition of ANXA2 may be a suitable anti‐cancer therapy (Wang et al., 2018).

## 10 ALIX

The apoptosis‐linked gene 2 (ALG‐2)‐Interacting protein X (ALIX) is one of the established protein markers enriched in exosomes (Vanessa et al., 2019). It is a cytosolic adaptor protein in mammalian cells, ubiquitously expressed, and concentrated in phagosomes and exosomes (Chatellard‐Causse et al., 2002; Odorizzi, 2006; Vanessa et al., 2019). ALIX was initially identified based on its association with the pro‐apoptotic signalling component ALG‐2 (Mahul‐Mellier et al., 2008; Missotten et al., 1999; Trioulier et al., 2004). Currently, we know that this protein has a pivotal role in regulating the endocytic membrane trafficking (Matsuo, 2004; Odorizzi, 2006), the virus entry process (Martin‐Serrano et al., 2003; Morita & Sundquist, 2004), the integrin‐mediated cell adhesions, the extracellular matrix assembly (Pan et al., 2008), and repair of the plasma membrane (Jimenez et al., 2014; Scheffer et al., 2014). This protein presents a tripartite domain organization consisting of an N‐terminal Bro1 domain, which is followed by coiled‐coils (CC) that build the V‐domain, and by a proline‐rich C‐terminal domain (Odorizzi, 2006). Bro1 mediates the localization to endosomes, while the proline‐rich region determines interaction promiscuity with proteins (Odorizzi, 2006). For instance, ALIX interacts with several ESCRT proteins in the formation of exosomes (Fujii et al., 2007; Odorizzi, 2006; Vanessa et al., 2019).

Iavello et al. evaluated the contribution of ALIX in the packaging of miRNAs in EVs released by human liver stem‐like cells (HLSCs) (Iavello et al., 2016). They found those EVs enriched in miRNAs eliciting antitumor as well as regenerative activity (miR ‐24, miR‐31, miR‐125b, miR‐99b, miR‐221, miR‐16, and miR‐451). Of relevance, authors highlighted the interaction between ALIX and AGO2 and the influence of relative miRNA abundance without affecting the number of released EVs (Iavello et al., 2016).

Overall, these observations indicated a synergistic activity of ALIX in determining the miRNA cargo into EVs. A dynamic analysis of its RNA‐dependent interactome might contribute to our understanding of the selective enrichment of EV‐miRNAs.

## 11 NUCLEOLIN (NCL)

Nucleolin (NCL), discovered in 1973, is a highly conserved RBP and one of the major nucleolar phosphoproteins in eukaryotic cells (Orrick et al., 1973). Three main structural domains form it: N‐terminal domain, enriched in acid glutamate/aspartate repeats, the central domain, containing four RRMs that mediate the interactions with nucleic acids, and the C‐terminal RGG region, that allows protein‐protein interaction (Figure 1). Acetylation and phosphorylation at N‐term domain contribute to its localization and RNA‐binding (Belenguer et al., 1990; Das et al., 2013; Ghisolfi‐Nieto et al., 1996; Salvetti et al., 2016; Ugrinova et al., 2018). NCL binds to RNA stem loop structures containing a UCCCGA consensus sequence (Bouvet et al., 2001) and associates with RNA oligonucleotides containing UUAGGG repeats more efficiently than the corresponding telomeric single‐stranded DNA (Ishikawa et al., 1993). NCL mostly localizes in nucleoli, and to some extent in nucleoplasm, cytoplasm, and cell membrane. NCL is involved in ribosome biogenesis, DNA repair and genome stability, cell division and survival, angiogenesis, and EMT. Its ability to form complexes with several different proteins could explain the pleiotropic function of NCL, still not completely understood (Birmpas et al., 2012; Jia et al., 2017; Ugrinova et al., 2018). It has been recently described as an antigen of cancer cells and cancer‐associated endothelial cells, mediating the biogenesis of miRNAs involved in tumour development, aggressiveness, and drug‐resistance. Of note, NCL post‐transcriptionally regulated miR‐21, ‐221, and ‐222, enhancing their maturation from primary transcript state (Pichiorri et al., 2013).

D'Avino et al. tested a novel NCL‐targeting immunoconjugate (4LB5‐HP‐RNase), engineered by fusion of the novel human anti‐NCL scFv 4LB5 and a human pancreatic RNase, on exosomes from derivatives of colorectal cancer cells (D'avino et al., 2016). Using miR‐21 as readout, their results suggested that, possibly, the binding disruption between NCL and miR‐21, mediated by 4LB5 scFv moiety, allows miR‐21 free‐form to be degraded by the RNase. More recently, Wang et al. designed a nanoplatform able to target NCL‐positive BC cells, exploiting the DNA aptamer AS1411 affinity to NCL. Reduced NCL expression levels and change of aptamer sequence impaired the binding efficiency (Wang et al., 2017).

NCL was found as the most abundant host cell natural protein within influenza virus‐like particles, VLPs, produced in suspension and serum‐free media by transient transfection of an inducible clone of HEK‐293SF cells (Venereo‐Sánchez et al., 2019). In the same work, NCL was not detected in EVs produced independently from non‐transformed HEK‐293SF cell line. The group, through this comparative study, identified NCL as a specific VLP marker to potentially guide VLP purification.

Since NCL functions are implicated in cancer, inflammation, and viral infection, the protein has been recently proposed as a druggable target. The first evidence of a small NCL inhibitor was identified in the anti‐tumour diterpene oridonin in Jurkat cells (Vasaturo et al., 2018). Moreover, NCL was validated as a target of curcumol in nasopharyngeal carcinoma (NPC) cells, through cellular thermal shift assay (CETSA), molecular docking (7.8 kcal/mol binding free energy) and cell‐based assay. The results revealed that the anti‐tumour effects of curcumol in NPC cells are mediated, at least partially, by NCL inhibition (Wang et al., 2018). A novel hybrid chalcone (3',4',5'‐trimethoxy‐5‐chloro‐isatinylchalcone, 3MCIC) interacts with different protein targets, confirmed by LAC (ligand‐affinity chromatography)‐MS, including NCL (Cao et al., 2016).

## 12 FUS/TDP‐43

Fused in Sarcoma/Translocated in Liposarcoma (FUS/TLS) is a predominantly nuclear RNA‐binding protein that belongs to the FET (FUS, EWSR1, TAF15) protein family together with EWS and TAF15RBPs. FUS contains an N‐terminal domain with a glutamine‐glycine‐serine‐tyrosine‐rich (QGSY‐rich) region, a highly conserved RRM domain, a zinc finger motif, and multiple RGG amino acid repeats at the C‐terminal (Figure 1). Analogously to TDP‐43, another ALS‐associated protein, FUS is a nucleo‐cytoplasmic shuttling factor working at transcriptional and post‐transcriptional levels. It has a role in DNA repair, transcription, RNA splicing, dendritic RNA transport, and miRNA biogenesis (Loughlin & Wilce, 2019). The binding of FUS to nascent pri‐miRNAs can recruit Drosha at chromatin sites of active transcription, promoting pri‐miRNA processing. In fact, in human neuroblastoma cells, the depletion of FUS reduced the level of several miRNAs, including miR‐9, miR‐125b, and miR‐132, which have important roles in neuronal metabolism and differentiation (Morlando et al., 2012). On the other hand, the over‐expression of FUS was directly associated with increased biogenesis of miR141 and miR200a (Dini Modigliani et al., 2014). Kapeli et al. demonstrated a stabilization effect of up to 330 mRNAs or degradation of 44 transcripts in human neural progenitor cells (Kapeli et al., 2016). Besides links with liposarcoma and myeloid leukaemia, this protein is involved in neurodegenerative disorders, such as ALS and frontotemporal lobar degeneration (FTLD) (Hortobágyi & Cairns, 2018).

Recent reports indicated an association between an imbalance in the nucleo‐cytoplasmic shuttling process and the presence of ribonucleoparticle aggregates in exosomes. Kamelgarn et al. detected FUS along with the interaction partners DDX3X and hnRNPA1 in the exosome fraction, and reported that the ALS‐associated FUS mutant R495X was present in a significantly higher level (Kamelgarn et al., 2016). Sproviero et al. showed that MVs of ALS patients were enriched with potentially aggregation‐prone FUS compared to control subjects. These observations lead to the hypothesis that EV‐carrying mutant FUS could promote the spreading of FUS‐mediated toxicity by a prion‐like mechanism (Kamelgarn et al., 2016; Sproviero et al., 2018).

Consistent observations in the same line of FUS, also the ALS‐associated TDP‐43 protein was reported to be present in exosomes from primary neurons of human amyotrophic lateral sclerosis brains (Iguchi et al., 2016) and plasma of ALS patients (Sproviero et al., 2018). Interestingly, the exposure of Neuro2a cells to those exosomes caused a cytoplasmic redistribution of TDP‐43, indicating a possible TDP‐43‐dependent propagation of proteinopathy through exosomes (Iguchi et al., 2016).

## 13 MVP (MAJOR VAULT PROTEIN)

Major Vault protein, also known as lung resistance‐related protein, is a ribonucleoprotein of 100 kDa constituting the main fraction (70%) of the multimeric vault ribonucleoproteins (Scheffer et al., 2000). The vault complex is composed of three proteins: MVP, vault poly (ADP‐ribose) polymerase (vPARP), and the telomerase‐associated protein 1 (TEP1), which binds to short non‐coding RNAs named vault RNAs (vRNA) (Berger et al., 2009). These large RNA–protein structures serve as intracellular shuttles for proteins and other small molecules between the nucleus and cytoplasm (Scheffer et al., 2000). MVP appears equally distributed in both nucleus and cytoplasm (Ryu et al., 2008). The MVP core of the vault complex is a polypeptide composed of proteins organized in a barrel‐like shape, with an invaginated waist and two protruding caps that can open in a flower‐like shape (Kedersha et al., 1991). MVP sequence consists of a ∼55 amino acid residue domain at the N‐terminal, which is the main repeat element in human MVP that folds in three‐stranded antiparallel β‐sheets, a central region and a coiled‐coil domain at the C‐terminal, which is fundamental for maintaining the structure of the Vault complex and the interaction with the other vault proteins (Kedersha et al., 1991; Kozlov et al., 2006; Van Zon et al., 2002). It is still unclear the mechanism underlying MVP ability to bind calcium ions, previously explained by the presence of three EF‐hand motifs in MVP sequence (Van Zon et al., 2002), later excluded following the most recent studies (Kozlov et al., 2006).

MVP is subjected to post‐translational modifications, being phosphorylated on Tyr residues after EGF stimulation, and is dephosphorylated by SHP‐2 (Kolli et al., 2004). Other studies also reported its interaction with the phosphatase PTEN, suggesting a role for MVP as a scaffold in signal transduction pathways (Kolli et al., 2004; Minaguchi et al., 2006). Importantly, MVP expression correlates with an increased multi‐drug resistance in cell lines, possibly mediated by its role in the intracellular transport of molecules. Notably, it is considered as a negative predictive marker of chemotherapy (Kickhoefer et al., 1998; Kong et al., 2000; Van Zon et al., 2001).

MVP was identified in exosomes derived from multiple colorectal cancer cell lines of (Xu et al., 2015), hepatocellular carcinoma and hepatocytes (He et al., 2015), ovarian cancer (Liang et al., 2013), neuroblastoma (Keerthikumar et al., 2015), melanoma (Peinado et al., 2012), bladder cancer (Welton et al., 2010), and nasopharyngeal carcinoma cells (Chan et al., 2015). Moreover, it has been found in exosomes isolated from human breast milk (Admyre et al., 2007), B cells (Buschow et al., 2010), thymus (Skogberg et al., 2013), platelets (Pienimaeki‐Roemer et al., 2017), saliva (Gonzalez‐Begne et al., 2009), and urine (Gonzales et al., 2009).

A sort of ‘a‐specific’ RNA‐binding activity of MVP has been observed in the study carried out by Statello et al. They reported a lower amount of total exosome‐derived RNA (of about 50%) upon MVP silencing. An RNA‐pull‐down assay validated the role of MVP on exosomes derived from HEK293 cells transfected with biotinylated full‐length MVP, whose RNA was later extracted and quantified (Statello et al., 2018). The relatively high abundance of RNA in exosomes as a function of MVP modulation indicated a potential crucial involvement of MVP in the sorting of miRNA and mRNA species into vesicles (Statello et al., 2018). The potential RNA‐binding selectivity of MVP has not been exhaustively investigated yet. An exception is a recent study which highlighted that MVP can sort miR‐193a inside exosomes (Teng et al., 2017). In detail, Teng et al. analyzed a distribution pattern of miRNA expressed by three types of exosomes isolated from primary mouse colon cancer, liver metastasis of colon cancer, and naive colon tissues. The metastasis‐derived exosomes carried the highest level of tumour‐suppressive miRNAs (miR‐193A), while the donor cells mostly expressed tumorigenic miRNAs. As further validation, MVP knockout caused miR‐193a accumulation in cells instead of exosomes, which lead to inhibition of tumour growth. These results suggested that the sorting of miRNA inside exosomes was not a passive phenomenon, but possibly mediated by RBPs, and that circulating exosomes could exert a sort of tumour suppressive effect on surrounding tissues (Teng et al., 2017).

Interestingly, a recent study on Zika virus showed that silvestrol, a natural compound of therocaglate family known to inhibit eIF4A (DEAD‐box RNA helicase eukaryotic initiation factor‐4A) was able to downregulate the expression of MVP in A549 cells (Elgner et al., 2018).

## 14 LIN28

LIN28, discovered in the nematode *Caenorhabditis elegans*, is an evolutionary conserved RNA‐binding protein of approximately 22 kDa, which in mammals has 2 paralogs: LIN28 (or LIN28A) and LIN28B. In vertebrates, these ribonucleoproteins share a high sequence identity (73%) and three conserved structural regions composed of an N‐terminal cold shock domain (CSD) and two knuckle‐type cysteine‐cysteine‐histidine‐cysteine (CCHC) zinc finger (ZnF) domains (Loughlin et al., 2012; Wang et al., 2016). LIN28A is mainly cytoplasmatic, while LIN28B is almost exclusively present in the nucleus and nucleoli (Balzeau et al., 2017; Piskounova et al., 2011). The family of Lethal‐7 (let‐7) microRNAs, involved in the regulation of differentiation pathways, is the main target of LIN28, which post‐transcriptionally suppresses the biogenesis of let‐7 precursors (pri and pre‐let 7) (Wang et al., 2012). LIN28 is a recognized stemness marker, essential to promote pluripotency with OCT4, SOX2, NANOG, and play a role in the regulation of de‐differentiation in both let‐7 dependent and independent manners (Tsialikas & Romer‐Seibert, 2015; Yu et al., 2007).

A single‐nucleotide‐resolution mapping of LIN28 binding sites, made by Ustianenko et al., showed that the presence of a (U)GAU sequence on RNA targets is necessary for their recognition by the CSD domain (Ustianenko et al., 2018), while ZnF domains bind to GGAG‐like motifs on the terminal loop of precursors of let‐7 miRNAs. Upon binding, it has been reported that the two LIN28 forms repress the maturation of let‐7 miRNAs with distinct mechanisms. Conserved GGAG/GGUG motifs located within exons and the 3′‐UTR of mRNAs were defined as binding sites with LIN28A/B, and multiple studies suggested that LIN28A/B might play a role in modulating the translation efficiency and splicing of target transcripts, although these mechanisms have not been fully characterized yet (Enriquez et al., 2015; Huang et al., 2012; Peters et al., 2016; Wilbert et al., 2012).

Several studies have shown that LIN28 proteins are overexpressed in different types of cancers (colorectal, breast, and ovarian), where the function of let‐7 as tumour suppressor against RAS or MYC is lost (Balzeau et al., 2017; Enriquez et al., 2015; Guo et al., 2006; Viswanathan et al., 2009; Wang et al., 2016). LIN28 proteins promoted tumour progression, the formation of metastasis and their overexpression correlated with a poor prognosis (Hamano et al., 2012; Viswanathan et al., 2009).

A recent study by Enriquez et al. investigated the oncogenic properties of exosomes secreted by ovarian cells overexpressing LIN28 (Alicka et al., 2019). An increased invasive phenotype was observed in HEK293 cells upon uptake of exosomes with high *LIN28* transcript levels (Alicka et al., 2019). In another study focused on genes involved in the control of the glucose–lipid metabolism, the expression of LIN28 transcripts was investigated in tissue‐derived mesenchymal stem cells (ASCs) obtained from patients with Type‐2 Diabetes (T2D) and compared with the content in deriving EVs. Also in this model, no evidence of LIN28 protein in EVs was reported, in contrast to its mRNA (Alicka et al., 2019). A recent work from McDaniel et al. investigated the potential therapeutic properties of liver stem cell‐derived EVs (LSCEVs) to treat cholangiopathies. They found a high expression of let‐7 miRNAs in LSCEVs (let‐7a and let‐7b), which suppressed the activity of their preferred downstream targets, as LIN28A/B expression decreased upon treatment of total liver isolates from multidrug resistance protein 2 (MDR2‐/‐) mice, with LSCEVs. In turn, LIN28 proteins reduced overall liver damage, acting on NF‐κB and IL‐13 signalling pathways, suggesting a therapeutic effect of LSCEVs in liver diseases mediated by the let‐7/LIN28 axis (Mcdaniel et al., 2019).

Among the inhibitors of LIN28 recently reviewed (D'agostino et al., 2019), a recent study investigated more on C1632 (N‐Methyl‐N‐[3‐(3‐methyl[1,2,4]triazolo[4,3‐b]pyridazin‐6‐yl) phenyl]acetamide), a compound already known to block the interaction between LIN28 and let‐7 (Chen et al., 2019; Roos et al., 2016). In particular, the authors found that C1632 was able to downregulate the programmed death ligand‐1 (PD‐L1) and inhibit the tumour growth in vitro and in vivo, suggesting that C1632 and its derivatives could be employed as multifunctional antitumor drugs (Chen et al., 2019).

## 15 SRP9/14

The signal recognition particle (SRP) is a cytoplasmic ribonucleoprotein complex, which in eukaryotes consists of six polypeptides (SRPs 72, 68, 54, 19, 14, and 9) and a 300‐nucleotide 7S RNA (RN7SL1) (Walter & Blobel, 1983). In Archaea, SRP19 and SRP54 represent the only two SRP‐constituting protein subunits (Bhuiyan, 2000). In eukaryotes, SRP9 and SRP14 bind to the 5’ and 3' terminal sequences of 7S RNA (47 and 39 nucleotides, respectively), forming the so‐called Alu domain (Weichenrieder et al., 2000). SRP RNA initially interacts with SRP9/14, SRP19, and SRP68/72 in the nucleolus, forming a pre‐SRP that is subsequently transported to the cytosol where it associates with protein SRP54 (Politz et al., 2000). At cytoplasmic level, SRP, via its conjugate receptor, is involved in targeting secretory proteins to the RER membrane in eukaryotes, or the plasma membrane in prokaryotes (Reyes et al., 2007; Römisch et al., 2006). SRP S domain recognizes the signal sequence of the ribosome‐associated nascent polypeptide, and the Alu domain promotes SRP docking of the ribosome‐polypeptide complex to the ER membrane (Hsu et al., 1995; Koch et al., 2003). RN7SL1 RNA catalyzes the SRP‐receptor interaction, directly mediated by conserved active site residues in the GTPase domains of both SRP and its receptor (Bradshaw & Walter, 2007; Keenan et al., 2001).

A few indications about SRP9/14 association in EVs were so far reported. Nabet et al. showed that stromal fibroblasts, triggered by breast cancer cells upon abnormal juxtacrine signalling, secrete exosomes with increased RN7SL1 RNA (Nabet et al., 2017). The group reported that exosomes mediate the RN7SL1 transfer from stromal cells to breast cancer cells: 5‐ethynyl uridine‐modification by azide‐linked fluorescein allowed to see the horizontal acquisition of stromal cell RNA, represented by transcripts regulated by POL3 besides RN7SL1 (White, 2011). The authors also showed that the SRP9/14:RN7SL1 RNA stoichiometry controls the accumulation of unshielded RN7SL1 in the cytoplasm and secreted exosomes.

A study published in 2019 introduced the *Scutellariabarbata* polysaccharides (SBPS) as potential SRP9/14 inhibitors in a hepatocellular carcinoma mouse model. After SBPS treatment at different doses, the examination of the serum proteins revealed reduced SRP9/14 as compared to its levels in tumour‐bearing mice (Li et al., 2019).

## 16 QKI

Quaking proteins (QKI) are spliced‐proteins encoded by the quaking gene (*QKI*) and part of an evolutionarily conserved class of RNA‐binding proteins called STAR (signal transduction and activation of RNA) proteins (Vernet & Artzt, 1997). The spliced isoforms encoded by *QKI* (QKI‐5, QKI‐6, QKI‐7, and QKI‐7b) are mainly expressed in the central nervous system (CNS), glial cells, oligodendrocytes, and neuronal progenitors (Larocque et al., 2009), where they act as regulators of brain development (Ebersole et al., 1996). In particular, QKI‐5 is mostly expressed during embryogenesis, while the other isoforms are found involved in the formation and maintenance of myelination (Zhao et al., 2010). These isoforms also differ in their subcellular localization and, while QKI‐5 is mainly located in the nucleus, QKI‐6, and QKI‐7 shuttle between cytoplasm and nucleus (Chénard & Richard, 2008).

Structurally, all STAR proteins share a tripartite domain of 200 amino acids composed by a single, large KH domain (maxi‐KH), surrounded by QUA1 and QUA2 domains (Vernet & Artzt, 1997) and Tyrosine residues in the C‐terminal region. The maxi‐KH and QUA2 domains are the regions involved in RNA binding, to RNA targets enclosing a 5'‐NACUAAY‐N (Barceló et al., 2014; Yáñez‐Mó et al., 2015)‐UAAY‐3' RNA recognition motif, while the QUA1 region facilitates the recognition of target RNAs by KH‐QUA1 domains (Galarneau & Richard, 2009; Ryder, 2004; Teplova et al., 2013). Multiple studies reported that QKI plays a central role as post‐transcriptional regulators of mRNA stability and export (Teplova et al., 2013) and alternative splicing of target messenger RNAs (Wu et al., 2002). QKI is involved in the regulation of the mRNA of major myelin structural genes, in particular, QKI affects the alternative splicing of MAG (myelin‐associated glycoprotein), inducing a demyelinating phenotype (Wu et al., 2002). It drives the nuclear export of MBP (myelin basic protein) mRNA in the myelin compartment preserving myelinogenesis (Ryder, 2004). Importantly, quaking mutant mice displayed a phenotypic pattern of neuronal dysfunctions resembling schizophrenia, explained by the lacking of expression QKI‐6 and QKI‐7 isoforms in oligodendrocytes of mutant mice (Galarneau & Richard, 2009; Hardy, 1998). Zhang et al. showed that the phosphorylation of Tyr‐rich ends of QKI hampers the MBP‐RNA binding activity of QKI (Zhang, 2003). In support of this study, a lower expression of QKI was found in the brain of 55 schizophrenia patients (Aberg et al., 2006). Other studies showed that QKI‐6 and QKI‐7 isoforms were promoters of oligodendrocyte differentiation and regulators of neural progenitors fate (Larocque et al., 2009). Alterations of the quaking gene in gliomas detected during a mutational screening on primary brain tumours also exist (Li et al., 2002) high expression of QKI in tissues of breast cancer patients correlated with shorter overall survival time, proposing QKI as a prognostic marker in synergy with SLUG, a pivotal player in the EMT process (Gu et al., 2019). Interestingly, QKI was identified as the principal regulator of biogenesis of circular RNAs during epithelial to mesenchymal transition (EMT) (Conn et al., 2015).

Studying EVs, the group of Wang et al. investigated the expression of miR‐208a and miR‐208b in vesicles released by hypoxic cardiomyocytes (CMs), mimicking the environment of hypoxia/reoxygenation (H/R) injury. They found an enrichment of miR‐208a and miR‐208b miRNA. Since QKI is known to inhibit the apoptosis of cardiomyocytes (CMs) and an in silico analysis predicted the binding site of miR‐208a and miR‐208b to 3’UTR of QKI, the authors tested the effect of this RNA‐RBP interaction by exposing hypoxic cardiomyocyte to EVs. Notably, the EV‐mediated transfer of miR‐208a and miR‐208b suppressed the protective influence of QKI on apoptosis, suggesting QKI as a target to regulate CM apoptosis (Wang et al., 2017).

## 17 TERT

Human telomerase reverse transcriptase catalytic subunit, TERT, is a ribonucleoprotein enzyme that is part of the telomerase complex, mainly involved in elongation of telomeres. Albeit ascribed as a ‘non‐canonical RBP’, the role of TERT is increasingly recognized in the binding of different RNA molecules (Bryan & Cech, 1999; Lingner, 1997). TERT interacts with the non‐coding RNA *hTR* that provides the template for the reverse transcription of TTAGGG repeats at the ends of chromosomes. The TERT subunit consists of a 127 kDa protein with an N‐terminal RNA‐binding domain and a C‐terminal RT‐like domain (Bryan & Cech, 1999; Lingner, 1997). Studies focused on *Tetrahymena thermophila* homolog evaluated TERT‐specific motifs, T1 and T2, as essential for the binding with two sequences of RNA component, hTR (nucleotides 1–209) and the Inserted Hairpin Element 1 (IH1), independently from the C‐terminal active site (Lai et al., 2001; Rouda & Skordalakes, 2007). Of relevance, the RBPs hnRNPF/H have been reported to regulate the activity of TERT upon binding to both enzyme and RNA components, contributing to cancer and human mesenchymal stem cell proliferation and senescence (Xu et al., 2020). In the nucleus, TERT can also act as a transcriptional co‐factor, as demonstrated for the Wnt/β‐catenin pathway member BRG1 (Yang et al., 2017) or the IL6, IL8, and TNFα cytokines (Ghosh et al., 2012).

As a consequence of cell stress, TERT also shuttles to the cytoplasm and mitochondria where it exerts non‐canonical extra‐telomeric functions (Chiodi & Mondello, 2012; Sharma et al., 2012). In particular, the overexpression of TERT coupled with the induction of reactive oxygen species (ROS) might trigger a translocation in the mitochondrial matrix where it could protect from oxidative stress, repairing stress derived mtDNA damage (Haendeler et al., 2009; Santos et al., 2006). Post‐translational modifications heavily influence the sub‐cellular localization of TERT (Seimiya, 2000).

The telomerase upregulation during cancer progression is one of the hallmarks of neoplastic transformation, as it sustains the increased rate of genome replication (Hanahan & Weinberg, 2000). Several studies have therefore investigated and proposed a role of TERT transcript as a serum biomarker in breast (Qi Chen et al., 2000), prostate (March‐Villalba et al., 2012), lung (Miura et al., 2006), gastric (Kang et al., 2013), colorectal (Terrin et al., 2008), and hepatocellular carcinomas (Miura et al., 2003). Especially in gastric and prostate cancer, the high level of TERT mRNA correlated with advanced stages of disease (Kang et al., 2013; March‐Villalba et al., 2012).

Skog et al. detected TERT mRNA in microvesicles derived from glioblastoma patients (Skog et al., 2008). More recently, Gutkin et al. detected this transcript in exosomes isolated from Jurkat (T cell leukaemia), MCF‐7 (breast carcinoma), K562 (chronic myeloid leukaemia), and HCT116 (colon carcinoma) cell lines. Remarkably, these vesicles were reported as able to induce the expression of TERT in telomerase‐negative fibroblasts and induce malignant phenotype (Gutkin et al., 2016). An exploratory study involving heterogeneous cohorts of cancer patients indicated that ∼60% of cases expressed detectable levels of *TERT* (Goldvaser et al., 2017).

The role of EVs as vehicle of TERT protein was addressed by Radeghieri et al., who tested primary amniotic fluid cells isolated from 25 subjects and found TERT in ∼30% of exosome samples (8 out of 25) (Radeghieri et al., 2017). More recently, the group detected TERT protein in rat bone marrow‐MSC‐derived EVs (Gissi et al., 2020). An interesting finding from Wang et al. associated telomeric repeat‐containing RNA (TERRA) fragments to EVs. TERRA results from the stress‐induced transcription of telomeric DNA repeats and is implicated in structured nucleic acid hybrids and ribonucleoprotein particles (Arora et al., 2014; Azzalin & Lingner, 2015). Cell‐free TERRA was co‐fractionated with human lymphoblastoid cell lines‐derived exosomes. Exosomal TERRA was able to induce the transcription of inflammatory cytokines in recipient peripheral blood mononuclear cells (PBMCs). A similar response was inducible with HCT116 exosomes engineered to express TERRA (Wang et al., 2015).

A broad panel of TERT inhibitors is currently available for telomerase targeting, ranging from small molecules, antisense oligonucleotides, immunotherapeutic peptides, or trans‐acting repressors (Ruden & Puri, 2013). GRN163L/Imetelstat is an established telomerase inhibitor consisting of a thio‐phosphoramidate TAGGGTTAGACAA oligonucleotide complementary to the template region of hTR element. It was reported as an efficient tool to interfere with the RNA‐binding activity of TERT (Gryaznov et al., 2007).

## 18 CONCLUSIONS

The data so far described indicate an important coverage of *qualitative* aspects, ranging from the mere detection of the protein to the inference of bound RNAs, linking RNA‐binding proteins to EV biology. As shown in Figure 1, a qualitative comparison of shared domain features among EV‐associated RBPs does not reveal enrichment of molecular clues preferentially involved in vesicular packaging. Nevertheless, a substantial proportion of hnRNP family members emerges as a well‐represented hub between the RNA post‐transcriptional control and vesicular trafficking. hnRNPs are well‐known factors involved in RNA metabolism, from maturation to translation, and the elucidation of their networks of protein:protein/RNA interactions could represent an interesting level of analysis in relation to the endosomal compartment. In different cellular contexts, caveolin‐1 was shown as a direct interactor of hnRNPA2B1 (Lee et al., 2019), while Rab27a was found modulated by hnRNPH1 (Datta et al., 2017), and hnRNPK directly involved in LC3‐depended EV loading and secretion (Leidal et al., 2020). Relevantly, other RBPs, such as YBX1 and MEX3C, were found in complex with central nodes of the ESCRT machinery (Hornung et al., 2020) or clathrin‐mediated endocytosis (Lu et al., 2017), respectively, underlying the existence of a significant role in the transport of, at least sub‐populations, of vesicles. These relationships gain mechanistic values when specific PTMs are associated with a cytoplasmic activity of the RBP and the EV‐RNA results consequently modulated. The sumoylation status of hnRNPA2B1 was found associated with enrichment of the protein in exosomes along with a plethora of miRNAs defined by a detectable motif (Villarroya‐Beltri et al., 2013). At the same time, its phosphorylation/O‐GlcNAcylation associated with a localization of the protein into distinct EV sub‐populations (Lee et al., 2019). Analogously, the accumulation of cytoplasmic YBX1 upon its phosphorylation induced secretion of proangiogenic factors in vivo (Gopal et al., 2015), in a context where the protein was revealed important in the vesicular packaging of abundant small ncRNAs (Shurtleff et al., 2016).

Several studies performed in different cellular contexts with case‐by‐case biochemical and statistical approaches, identified some vesicle‐enriched RNA motifs variable in lengths and complexity. The EV‐RNA consensus at a glance could propose sequence subsets recognized by different RBPs, such as the A/G‐rich stretches by hnRNPA2B1, hnRNPH1, hnRNPQ, AGO2, and ANXA2, AU‐rich elements by HuR and hnRNPC1; C‐rich stretches by hnRNPG, hnRNPK, YBX1, and NCL. These subsets can find consistency from the reported, experimentally validated protein:protein interactions, where the RNA mediates these networks. These observations indicate that RNA packaging into EVs might significantly be impacted by a competitive interplay of RBPs. Sparse data on AGO2 protein could represent just an example of a dynamics where the cooperation with other RBPs and the endosomal compartment appeared in some works crucial to convey different miRNAs inside EVs (Gibbings et al., 2009; Mckenzie et al., 2016). Transcribed precursor miRNAs are processed by Drosha complex, then exported into the cytoplasm and digested by Dicer complex to reach maturation. As recently reviewed (Zhang et al., 2015), four potential mechanisms of miRNA sorting into EVs have been described: *(i)* The neural sphingomyelinase 2 (nSMase2)‐dependent pathway; *(ii)* The sumoylated hnRNPs‐dependent pathway; *(iii)* The 3′ miRNA sequence‐dependent pathway; *iv)* The AGO2/miRISC‐related pathway. Besides the relative dosage/equilibrium of single miRNAs that could be indirectly co‐regulated by messenger RNAs and potentially described by the competing endogenous RNA (ceRNA) model (Salmena et al., 2011), the contribution of the phospholipid bilayer of forming vesicles has been recently described as part of the RNA selection mechanism, where the fluid membrane environment can electrostatically favour specific RNA sequences, as also demonstrated in terms of the affinity of different RNA EXOmotif sequences (Janas, 2006). In this frame, the interactions of RNAs with raft‐like regions of the lipid bilayer, for instance influenced by sphingomyelin, cholesterol, and ceramide composition, could be part of a mechanism contributing the RNA sorting into vesicles. Intriguingly, the connection of RBPs with the phospholipid bilayer, as already demonstrated for hnRNP or AGO2 proteins (Villarroya‐Beltri et al., 2013), could significantly elucidate the mechanism of trafficking and functional delivery of small non‐coding RNAs from the nucleus to secreted vesicles.

In cancer, the observed up‐regulation of most RBPs here described was found to induce an altered post‐transcriptional control, which could, in turn, generate transcript unbalance in the secreted vesicles. When mechanistically validated, this paradigm provides the rationale to search for specific RNA sequences in biological fluids and EVs to investigate them as potential biomarkers. Interestingly, Xu et al. detected higher levels of *hnRNPH1* mRNA in vesicles from the serum of hepatocellular carcinoma patients (Xu et al., 2018), in a context where an aberrant expression of hnRNPH1 was reported in different solid tumours, including hepatocellular carcinomas. Therefore, EVs could offer at least two distinct layers of investigation by including the detection of RBP mRNAs and RBP‐bound transcripts in a liquid biopsy test.

However, the connection of RBPs’ activity with EV biology requires comparative studies based on *quantitative* characterizations of protein localization, dosage, and RNA‐binding efficiency both at the intracellular and vesicular levels. Nevertheless, systematic EV isolation, characterization, and RBP/RNA detection methods could be successfully integrated with a frame of normalization‐based approaches to describe the biological matching between cells and secreted EVs, and among EVs recovered from different sources. These approaches should also include strategies to get insights on secondary RNA structures that could lead to discovering non‐obvious, key *trans*‐acting factors. In this view, we could elucidate a potential ‘asymmetric relevance’ of RBPs and identify new mechanisms to explain and predict the outcomes of EV‐RNA content from the vesicular transport.

Table 2 summarizes the EV isolation methods and biological indication in EVs, the post‐translational modifications (PTMs), main RBP interactors, RNA motifs, and includes identified RBP small molecule inhibitors. Some of these inhibitors could represent potential tools that could be successfully used as complementary approaches to study an RNA‐packaging effect into EVs.

## CONFLICT OF INTEREST

The authors declare no conflicts of interest.
